# The chemical and attitudinal differences between commercial and artisanal products

**DOI:** 10.1038/s41538-019-0053-9

**Published:** 2019-09-06

**Authors:** Cecilia T. Cirne, Michael H. Tunick, Rosemary E. Trout

**Affiliations:** Department of Food and Hospitality Management, Drexel University, 101 N. 33rd St., Philadelphia, PA 19104 USA

**Keywords:** Science, technology and society, Chemistry

## Abstract

Here we report a study of artisanal grain, coffee, ice cream, cheese, and chocolate made in the Philadelphia and New York areas, exploring the chemistry responsible for the differences between artisanal and mass-produced food, the rationale that artisans have toward making their products, and consumer attitudes toward purchasing artisanal food. The contrasting techniques used in manufacturing these two classes of food lead to differences in composition, flavor, and texture. Dairy products made from pasture-fed cows, for instance, display more complex flavor profiles owing to the greater variety of plants the animals consume. Consumers are willing to pay more for artisanal food, feeling that it tastes better, is healthier, and helps support family-owned operations. Producers not only want to be able to control their businesses, but also wish to create better and more-authentic food in an environmentally friendly manner. The psychology surrounding artisanal food is partly based on their chemistry.

## Introduction

The Division of Agricultural and Food Chemistry of the American Chemical Society hosted a highly successful symposium on artisanal foods at the ACS Spring National Meeting in 2017. As a follow-up to that symposium, a study of several types of artisanal foods was conducted. Artisanal products have become increasingly popular in recent years, and the word “artisana” can be found plastered on packaging in every supermarket, corner store, and farmers market, even on foods that are definitely not artisanal.^1^ This surge in popularity comes from the growing support for the social and environmental issues behind artisanal foods, such as sustainability, traceability, and the commitment to everything local. Unfortunately, there is a lack of definition when it comes to artisanal products. The term is so widely used and there seems to be commonalities between artisanal products across all sectors of artisanal foods. Some of these include small batch, handmade, locally sourced, and natural. For this research, several local companies were visited, manufacturing processes viewed, employees and owners interviewed, and customers surveyed to gather as much information as possible. The food categories selected—grain, coffee, ice cream, cheese, and chocolate—are produced artisanally in the Philadelphia and New York areas by people who allow visits to their plants. The primary difficulty when talking about artisanal products from an industry standpoint is that there are no guidelines or regulations for “artisanal”. Because of this, artisanal has become a vague, overused term that has begun to lose its meaning.

From all of this research, along with review of the available literature (which is scant), specific differences between artisanal and commercial products were discerned. These differences occurred in preproduction, production, and postproduction. Some of the differences are chemical, whereas others are differences in producer and consumer attitudes about the company ideals and products created. These differences show that there are some unsaid, but respected and common characteristics of artisanal products that could eventually lead to a Standard of Identity from the U.S. Food and Drug Administration, which could give more clarity on the term “artisanal”.

## Results and discussion

### Grains

Several differences exist between the production and manufacturing of commercial grains and artisanal grains, including sourcing, machinery, milling types, and good manufacturing processes. To label a product “stone ground”, it has to be passed over a milling stone before it is packaged. This does not always mean that the grains were ground by milling stones to create flour; in some cases the already-produced flour, which has been roller milled, is passed over a stone and called stone ground. One chemical difference that results from either roller milled grains or stone ground grains is the nutritive values of the products. In roller milled grains, it is becoming more and more popular to use pearled grains, which have the bran removed before milling.^2^ The entire grain is often used in true stone ground products, including the bran. It is important to note though that the term stone ground is not mentioned in FDA regulations. In this study, the stone ground products were examined at Castle Valley, an artisanal mill noted for its stone ground grain products. Depending on how the bran is handled in relation to the final product, the nutritive value of the grain and its subsequent products, such as flour, changes. One example is the changes in important non-nutrient antioxidant phenolic compounds. In stone ground, whole-grain products, phenolic compounds are found in high quantities because the bran is fully incorporated into the flour from the beginning of the process. In commercially produced grains, the phenolic compounds varied with the grain and the amount of bran within each product. For example, one study found that wheat bran contained 4527 mg phenolic acids/kg but white wheat bread contained only 111 mg/kg, as white flour does not contain any of the bran.^3^ In another study, phenolic compounds in pearled, roller milled grains were tested. With each pearling, more of the bran was removed and consequently the amount of phenolic compounds decreased. The range in phenolic levels of the pearled and roller milled wheat was 1300–5300 mg/kg^2^. The most-significant variations in the amount of phenolic compounds, however, resulted from sourcing and locations. This is another big difference in commercial and artisanal grains. Artisanal grains are sourced locally from smaller farmers, whereas commercial grains can be sourced from all over the country if not the world. Many times commercial grains come from large commodity farms where grains are bred for yield over quality and the growing conditions are not at peak because of frequency and nutrient depletion in the soil. Artisanal grains are bred more for flavor and specialty in richer soil but are grown in small batches with less of an emphasis on yield. This difference in growing and sourcing changes the nutritive quality of the grains, which is only further changed by the milling process chosen.

Another difference when addressing artisanal and commercial grains is the attitude of both manufacturers and consumers. When speaking to the co-owner of Castle Valley, Mark Fischer, he described the grains that he works with to create their whole berry, whole grain, and bolted flours, as “living food”, which changes with time, humidity, temperature, and season. Fischer is committed to the idea of his product being biologically active that he makes sure all dead and compromised grains are removed before the milling process. He sources all of his grains from small specialty farms within a 60 km radius, which breed grains specifically for Castle Valley. Mark and his wife Fran take immense pride in the quality of their product, from start to finish, which reflects in how their consumers perceive their products. According to the Fischers, they have a range of consumers, from restaurants, retail outlets, and individuals, but the commonality is the support of a locally grown, cleanly processed, high-quality, flavorful, nutritious product. They have had several reports of consumers with celiac disease and various degrees of gluten intolerance enjoy their products with no negative health consequences or reactions. Other consumers, especially international individuals, buy and use Castle Valley products because they are reminiscent of the natural, homemade flours from other countries. One example of this was a Pakistani group who became emotional over the products because they were finally able to make traditional flatbreads that are not possible with commercial enriched white flours. Consumers who prefer commercially produced flours and grains prioritize convenience and cost, whereas consumers who prefer artisanal products, like those of Castle Valley, prioritize flavor, nutrients, and local support.

### Coffee

Coffee is a growing area in the food industry, with artisanal specialty coffee becoming more popular. Coffeehouse chains such as Starbucks were founded in the United States in the 1980s, which led to growth of small-batch artisanal coffee roasters and independent or small-chain coffeehouses that may have direct relationships with the growers.^4^ La Colombe, a company that has an artisanal sector and a more commercial sector, was visited. There are several differences between mass-produced commercial coffee and artisanal coffees, which starts with the sourcing of the beans. One of the greatest factors when it comes to artisanal coffee is the traceability of the beans. When commercial blends are made, beans are purchased from several different countries and they are made to be a consistent blend, regardless of their source. Artisanal coffee on the other hand is usually about traceability. Most artisanal coffees are single origin or a seasonal blend from two different countries, but they are all based on individuality and availability. These roasts usually have much deeper flavor notes that include dark chocolate, nuts, or fruits. In artisanal coffees, the variances in product are preferred and embraced unlike commercial blends.

Another contrast between commercial and artisanal coffees is the treatment of the beans, specifically in the roasting process. Commercial roasting is done with speed as the primary concern, which is usually ~ 7 min at ~ 210 °C but depends on the specific roast. Artisanal roasting is done with flavor as the primary concern, which usually results in a roasting time of 10.5–15 min.

The other large difference between commercial and artisanal coffee is the processing. While machinery is involved in the processing of each, artisanal coffee is small batch and usually involves doing more by hand while commercial processing is almost completely reliant on machinery. For example, in the artisanal section of La Colombe, all of the packing is done by hand while in their commercial section it is all automated.

### Ice cream

Within the ice cream industry, there is a range in sourcing and production that creates either an artisanal or a commercial product. These two facets of ice cream have several differences that start as early in the process as the milk used. Three artisanal producers, Little Baby’s, Weckerly’s, and Franklin Fountain, were visited. One major difference between them was in the material used for the ice cream base. Little Baby’s is an artisanal ice cream company based in Philadelphia, but they are on the larger end of the artisanal spectrum, having a handful of shops as well as pints for retail. Although they do have a close relationship with the farm where they get their base, Trickling Springs, Little Baby’s uses a pasteurized, pre-blended base containing gums and stabilizers, which is more characteristic of commercially produced ice cream. Weckerly’s and Franklin Fountain, on the other hand, are true small batch, locally sourced, handmade artisanal ice cream companies, each with one shop in Philadelphia. Weckerly’s does not use a predetermined ice cream base, but rather use unprocessed raw milk from Seven Stars dairy farm. This extra step allows for complete control of the product, from beginning to end. Instead of buying a premade mix, Weckerly’s is able to create their product completely from scratch with ingredients that are 100% traceable. They are also able to ensure that their products are free of gums and stabilizers. Franklin Fountain mixes their own product as well, using pasteurized milk.

Overrun, the amount of air incorporated in ice cream differs between artisanal producers, who use a lower percentage of air, than industrial producers. Artisanal ice cream is typically produced using fresh ingredients and do not include additives such as stabilizers.^5^

In terms of flavors, these producers use keep seasonality in mind, which means that not all flavors are available year round. This is a key difference between commercial and artisanal ice cream. Although many commercial producers have seasonal flavors, they are a very small percentage of the total products made. With artisanal ice cream, seasonal flavors are the majority, if not the entirety, of the company’s products. This seasonality is not only important in differentiating products and attracting a specific consumer base but also in the ethics of the company. The three artisanal producers stressed the importance of sustainability and environmental ethics, which directly relates to the availability and seasonality of specific fruits and herbs. A related specialty of artisanal ice cream is the variation in flavors and deviation from the “norm” or the “classics”. Many of the flavors presented at these shops are twists on classics, such as Bourbon Vanilla or Dominican Fair Trade Cocoa Chocolate, or completely different and creative flavors, like Blueberry Rum or Cherry Hibiscus. These types of flavors are not suitable for large commercial producers because they are more expensive to produce and not as widely consumed as Cookie Dough or Mint Chocolate Chip. One interesting difference between commercial and artisanal producers, and also between Little Baby’s and Weckerly’s, is consistency within flavors. Little Baby’s and Franklin Fountain, like commercial producers, value consistency year round no matter the variations in their base ingredients. Weckerly’s on the other hand “embraces inconsistency” within their ice cream. The milk that they receive changes based on season and diet, which means that the product resulting from it changes throughout the year.

### Cheese

Artisanal cheese has become more popular over the last several years, and is growing a larger consumer base. Cherry Grove Farm in Central New Jersey was visited as part of this study. One main difference in artisanal cheese making is the treatment of cows and quality of milk. Cherry Grove has 80 dairy cows that are allowed to graze around the farm. They are artificially inseminated for the purpose of maintaining good health and controlling genetics. This causes higher quality milk that varies by seasonality by relaxed cows that have a controlled atmosphere and genetics. Starting with a high-quality base product creates a higher quality final product. In addition, artisanal cheeses contain no additives such as food coloring or stabilizers.

Another aspect of the milk that varies within artisanal and commercial cheese is nutritional value. Seasonality affects the components of milk and artisanal cheesemakers often do not correct for the differences. In a comparison of milk from cows consuming conventional feed and those on an organic regimen, the organic milk had higher levels of conjugated linoleic acid and α-linolenic acid (the major omega-3 fatty acid in milk), and less stearic and linoleic acid (the major omega-6 fatty acids in milk) during the spring–summer grazing season.^6^ The mineral contents varied much more than the macronutrients, but organic milk still had the overall highest numbers. When there are fewer additives in cheese, the milk used becomes more and more important in the final product. Varieties in makeup of the milk change the flavor and composition in the artisanal cheese made.

Large commercial operations subject their milk to high-temperature short-time pasteurization (72 °C for 15 s), but small-scale cheesemakers use vat pasteurization (63 °C for 30 min) or none at all. Those who do not pasteurize must age their cheese for ≥60 d to insure that any pathogens present will have died off or been reduced to a safe level before the cheese is sold.^7^

Raw milk cheese contains the bacteria naturally found in milk. Various species of Enterococcus, Lactobacillus, Lactococcus, Leuconostoc, and Streptococcus have been identified in Mexican raw milk cheese, and many of these bacteria may play roles in flavor development.^8^ A few of these species are used in starter cultures that are deliberately added to milk shortly after the start of the manufacturing process of most cheese varieties. The increased and broader presence of bacteria in raw milk cheese allows for faster and more- intense ripening, which increases proteolysis and lipolysis. The types and levels of flavor are elevated and the texture is different from corresponding pasteurized milk varieties.

Artisans often age their cheese on wooden shelves, which impart additional flavors from the wood surface, retains some microbes from the cheese surface, and helps regulate humidity in the aging room. They release the cheese for sale when they feel the flavor has reached an optimum. In a study of commercial and artisanal Manchego cheeses from Spain, 12 mo of ripening artisanal samples resulted in sensory characteristics that were significantly different from those ripened for shorter times. The olfactory aspects of the industrial cheese samples were similar at all ripening times.^9^

### Chocolate

Artisanal chocolate has become one of the most popular food trends in the past several years. The concept of terroir, the sense of place that includes growing and processing conditions that give a food its unique character, has spread from wine and cheese to other foods such as chocolate, where the origin of the beans gives a producer an opportunity to express its identity.^10^ Three different chocolate companies were visited: Fine & Raw, Brooklyn Born, and Raaka, all of which are artisanal. Like coffee, sourcing is one of the biggest differences in commercial and artisanal chocolates. Most artisanal companies, including Raaka and Fine & Raw, are bean-to-bar. Fine & Raw sources the majority of their heirloom beans from organic, sustainable farms in Ghana and Ecuador. Raaka sources their organic beans from the Dominican Republic, Tanzania, and Peru. Brooklyn Born on the other hand uses an Ecuadorian Peruvian blend of chocolate liquor, not beans. Most commercial companies use various blends of chocolate liquor from Western Africa.

The nutritional labels on a number of 70% cocoa chocolate bars were compared to determine the nutritional differences between commercial and artisanal companies (Table 1). Serving sizes ranged from 12.5 to 40 g, but the data were converted to 40 g samples. The labels were mostly similar; the majority of differences were in the ingredients, including dairy and different types of sugars, from coconut sugar, unrefined cane sugar, and processed sugar. Compositional similarities between artisanal and commercial products were also noted in grains, coffee, ice cream, and cheese, indicating that differences in flavor and texture, which do not appear on nutritional labels, are key to determining preferences.

**Table 1:** Nutritional and compositional differences between artisanal and commercial chocolate

	Artisanal chocolate (averaged)	Commercial chocolate (averaged)
Serving size	40 g	40 g
Calories	230	230
Sugar	11.4 g	11.3 g
Sugar type	Organic cane sugar/organic coconut sugar	Sugar
Fat	15.7 g	18.4 g
Fat type	Organic cacao butter	Cocoa butter

### Attitudes

When surveying customers and asking about their attitudes toward artisanal products, there were two major difficulties and three major reasons to buy artisanal food. The two difficulties are price and availability. Most artisanal products cost more than commercial ones and they are less-widely available, which make them much less accessible. In a culture based on convenience and ease, accessibility becomes a huge challenge for consumers and the growth of a small business.

The three major driving forces in purchasing artisanal food are flavor, health, and ethical responsibility. Taste has always been and will remain one of, if not the most, important factor when choosing any food. Many consumers found that the taste in artisanal was better and therefore justified whatever difficulties there are in buying them. Health concerns are also a growing issue in any and all aspects of the food industry. For years, food companies have been trying to decrease fat, cut out sugar, and lower caloric intake. More recently, health-based diets, such as vegetarianism/veganism, gluten free, and organic, have surged in popularity and sales. All of the artisanal companies visited, whether for marketing or ethics, had a number of certifications and nutritional claims. Ethical responsibilities range from company to company but they include responsible farming and sourcing, job security, close and direct relationships, donating and supporting to nonprofits, community support, environmental consciousness, and sustainability. Although these three reasons are unanimous, certain reasons are more prominent in specific products. For example, in chocolate, ice cream, and cheese, flavor is the driving force. In grains, health was much more prominent than any other sector. Chocolate and coffee also saw the highest responses related to sustainability and environmental ethics. Consumers respond to companies within a cause or commitment to better health, the environment, or aspects of society and feel a sense of responsibility to do good deeds. The effort exerted by artisans and the uniqueness of their products have also been cited in consumer purchasing factors.^11^

Consumers in a study about attitudes toward artisanal cheese spoke about social information like the “ethos of craftsmanship” and “farm story”. It was important that the cheese was “trying to be” something, as opposed to being perfect but valueless. Consumers believe they can sense this struggle to express value through the cheese and see it contribute positively to the sensory experience of the cheese. The authors concluded that consumers combine information about producer practice, social context, and the materiality of the product through an active, learned practice of sensory perception.^12^

Artisans often speak about being nurturing and more reactive to the raw materials, sometimes treating them as living substances. They are inclined to use traditional methods that have been in place long before mechanization, but they also experiment with recipes, procedures, etc., in order to create a new variety that they can call their own. A descriptor used independently by cheesemaker Stefanie Angstadt and ice cream maker Eric Berley is “storytelling”—relating the journey from growing to final product. “Back to the land”, *terroir*, hands-on, craft, and “doing what I love” are terms often deemed important by these artisans.

## Methods

This research started with several onsite visits to a number of companies across New Jersey, New York City, and the Greater Philadelphia Area. While on these onsite visits, owners and employees talked about their companies. In total, nine different companies were visited, comprised of one mill, one dairy farm, three ice cream companies, three chocolate companies, and one coffee company. They were asked why they went into the business they did, their commitment to an artisanal or commercial sector, their consumer base, personal attitudes towards the business, and their manufacturing processes. In some cases, the opportunity was given to get a guided tour through of the processing facilities and see firsthand the inner workings of each business. Chocolate melting, coffee roasting, and cheese making were observed up close.

In addition to onsite visits and employee interviews, consumer surveys were performed on attitudes regarding artisanal and commercial products. These surveys were conducted with customers at the farmers markets at Corinthian and Fairmount Sts. in Philadelphia and in Rutherford, New Jersey, Whole Foods Callowhill St. location, and Philadelphia’s Reading Terminal Market. Consumers were asked about why they choose foods marketed as artisanal, their opinions on certifications, like Organic or Gluten Free, the difficulties with artisanal and commercial, and ultimately their definition of artisanal foods.

## Data Availability

Data available on request from the authors.
